# Predictors of low birth satisfaction among Iranian postpartum women: A cross‐sectional study

**DOI:** 10.1002/nop2.1104

**Published:** 2021-10-30

**Authors:** Forough Mortazavi, Maryam Mehrabadi

**Affiliations:** ^1^ Non‐Communicable Diseases Research Center Sabzevar University of Medical Sciences Sabzevar Iran; ^2^ Health Chancellery Sabzevar University of Medical Sciences Sabzevar Iran

**Keywords:** birth satisfaction, cross‐sectional study, Iran, postpartum, predicting factors, women

## Abstract

**Aim:**

To investigate predictors of low birth satisfaction in a sample of Iranian postpartum women.

**Design:**

This was a cross‐sectional study.

**Methods:**

This study was conducted on 767 postpartum women using a convenience sampling method between June and September 2019. We collected data on socio‐demographic variables, maternal well‐being, fear of childbirth and birth satisfaction. We used multiple linear regression analyses to determine predictors of low birth satisfaction.

**Results:**

The women who gave birth by elective caesarean, emergency caesarean and vaginal birth were 13.2%, 19.06% and 67.8%, respectively. Predictors of low birth satisfaction were primiparity [Beta = −0.131, CI (−2.745, −1.021)], low level of well‐being [−0.119, (−2.514, −0.844)], low [−0.193 (−5.052, −2.568)] and moderate [−0.143 (−2.999, −1.199)] satisfaction with pregnancy, moderate satisfaction with spouse's emotional/financial support [−0.150 (−3.718, −1.595)], emergency caesarean [−0.086 (−2.713, −0.360)], severe fear of childbirth [−0.315 (−5.701, −3.911)] and long interval between admission to hospital and giving birth [−0.188 (−0.233, −0.119)].

## 1 INTRODUCTION

Childbirth is a unique and powerful experience in a woman's life. Childbirth dissatisfaction reduces maternal desire for subsequent pregnancies (Rashidian et al., 2019) or increases the interval between them (Gottvall & Waldenstrom, 2002). In previous studies, several variables have been found to be associated with childbirth satisfaction including planned pregnancy, planned childbirth (Tadele et al., 2020), women's knowledge of labour, low‐intensity labour pain (Jafari et al., 2017), short labour (Fumagalli et al., 2021), multiparity, women's participation in maternal care decision‐making process (Takacs et al., 2015), giving birth in primary level facilities (Mocumbi et al., 2019) or in private hospitals (Karoni et al., 2020) and empathy of midwives/physicians and respectful behaviour of caregivers (Pantoja et al., 2020; Takacs et al., 2015).

In recent decades in Iran, increased maternal request for caesarean (Darsareh et al., 2017) and its high rate (Gibbons et al., 2010) has turned into a challenging public health issue. At the same time, the total fertility rate (TFR) has been declining since 1992, falling to 1.8% in 2005 and remaining close to that level since then (Cincotta & Sadjadpour, 2017; Karamouzian et al., 2014). These developments have prompted health officials to explore policies that may reduce the rate of caesarean birth and encourage women to have more children. In 2014, a new policy initiative called the Health Transformation Plan (HTP) was launched in the Iranian health system (Moradi‐Lakeh & Vosoogh‐Moghaddam, 2015). The HTP consisted of many features and interventions such as improvement and renovation of maternity care facilities, cost‐free normal delivery, free prenatal education classes and allowing pregnant women to have a doula in labour and birth (Moradi‐Lakeh & Vosoogh‐Moghaddam, 2015; Setudezadeh & Yousefinezhadi, 2019). At the same time, women's satisfaction with childbirth has received growing attention from health officials and hospitals have been required to assess maternal satisfaction with the received care and services periodically (Yousefinezhadi et al., 2017). Unfortunately, few studies have been performed in Iran to evaluate maternal birth satisfaction as one of the main policy goals of the HTP and other attendant measures.

## 2 BACKGROUND

In recent decades, a number of factors have led to demands for quality care and women's rights to receive respectful maternity care during labour and birth. Among these factors, we can point to economic growth, rising living standards and the decreasing maternal mortality rates in almost all countries. In response, health researchers and policy makers have turned their attention to measures which could contribute to a positive experience of childbirth. Respectful maternity care may lead to maternal birth satisfaction (White Ribbon Alliance, 2011). Dissatisfaction with natural childbirth may also increase maternal request for elective caesarean (Johnson & Slade, 2002).

The Birth Satisfaction Scale – Revised (BSS‐R), developed in 2014 by Martin and Martin, has been recommended as the measure of choice for assessing women's experiences of childbirth by the International Consortium for Health Outcome Measures (ICHOM) (Martin & Martin, 2014). The scale has been translated and validated in different languages, including Italian (Nespoli et al., 2018), Turkish (Goncu Serhatlioglu et al., 2018), Slovak (Skodova et al., 2019) and Spanish (Romero‐Gonzalez et al., 2019). The Birth Satisfaction Scale – Revised contains three factors: stress experienced during labour, women's personal attributes and quality of care provision. We believe that psychological factors such as fear of childbirth and psychological well‐being may have a role in maternal birth satisfaction. However, only few studies have investigated the effects of psychological factors on maternal birth satisfaction.

Fear of childbirth is a common problem in both developed and developing countries with significant impact on women's well‐being (Nilsson et al., 2018). It is also an important psychological factor influencing maternal birth satisfaction (Ghanbari‐Homaie et al., 2021). One of the most widely used scales measuring fear of childbirth is the Wijma Delivery Expectancy/Experience Questionnaire (W‐DEQ) (Martin et al., 2020). The W‐DEQ was designed in 1998 for the purpose of assessing fear of childbirth in antepartum and postpartum periods (Wijma et al., 1998). The scale showed excellent reliability (Cronbach's alpha =0.93). The moderate correlations between Wijma scores and the scores from other scales confirmed the validity of W‐DEQ (Wijma et al., 1998). The W‐DEQ cut‐off point of 85 is used for screening women with severe fear of childbirth (Toohill et al., 2014).

In previous studies, maternal mental health and well‐being have been mostly investigated as something which is an effect of childbirth experience and not as a factor which could impact positive experience of childbirth. For instance, we can refer to the results of two studies which indicate that positive birth experience could predict postpartum mental health (Havizari et al., 2021; Hutchinson & Cassidy, 2021). The World Health Organization's Well‐Being Index (WHO‐5 Well‐Being Index) assesses the well‐being of individuals over the preceding two weeks. It is a short instrument designed to assess the level of emotional well‐being and consists of five positively worded items ("About the WHO‐5.", 2020). A cut‐off point of 50 has been established for use in screening individuals for depression. The Persian version of the scale which has been validated in pregnant women can be used to assess mothers’ well‐being.

Considering that birth satisfaction influences women's decisions about future pregnancies and their request for caesarean, we aimed to identify factors associated with low birth satisfaction in a sample of Iranian postpartum women. This is the first step in adopting proper strategies to promote women's birth satisfaction.

## 3 METHODS

### 3.1 Design, participants and data collection

This cross‐sectional study was performed using data which had been collected for the validity study of the Persian Birth Satisfaction Scale (the Persian BSS‐R) (Mortazavi et al., 2020). Our study population comprised women who had given birth and had been transferred to the postpartum wards of Mobini Hospital, operated by Sabzevar University of Medical Sciences, Sabzevar, Iran. Women filled out the questionnaires during the first 24 hr after giving birth. The sampling in our study was performed by convenience sampling method in the hospital and lasted from July to September 2019. The average annual birth rate in the hospital is 6,000. The inclusion criteria were having a healthy newborn baby, reading literacy and physical ability to fill out the questionnaires. We excluded women with severe mental illness and those with severe postpartum complications. We placed the three questionnaires in a folder, and two graduate midwives distributed the written consent forms and the folder among the participants who had given their verbal consent to take part in the study. Then, the socio‐demographic and obstetric information of mothers was collected from their medical files and was recorded in separate questionnaires. All the questionnaires were anonymous.

### 3.2 Instruments

#### 3.2.1 Interview form

The women completed a two‐section questionnaire. The first section contains questions on socio‐demographic characteristics. The second section consists of obstetrical information (such as parity, mode of birth, induced or spontaneous labour pain, pain relief method during labour, having a doula at birth). The women also rated their level of satisfaction with pregnancy, spouse's emotional/financial support and conjugal and sexual satisfaction on a five‐point Likert scale ranging from 1–5 (1 = not satisfied to 5 = very satisfied). With regard to the satisfaction with pregnancy, we asked the following question: Given the health problems you faced during your pregnancy, how satisfied are you with your pregnancy? (Data S1).

#### 3.2.2 The Birth Satisfaction Scale – Revised (BSS‐R)

The Birth Satisfaction Scale – Revised (BSS‐R) consists of ten items that participants respond to on a four‐point Likert scale which ranges from 0–4 (0 = strongly disagree to 4 = strongly agree). The minimum and maximum scores of the questionnaire are 0 and 40, respectively. The reliability of BSS‐R has been confirmed (Cronbach's alpha = 0.79). The scale was translated into Persian (Persian BSS‐R) in 2019, and its validity and reliability were investigated. The results of the confirmatory factor analysis on the scale confirmed the structure proposed by the scale developers. The reliability of the Persian BSS‐R has been confirmed (Cronbach's alpha = 0.76) (Mortazavi et al., 2020).

#### 3.2.3 Wijma Delivery Expectancy/Experience Questionnaire (W‐DEQ) version B

The W‐DEQ has a single factor with 33 items. Each item is scored on a six‐level Likert scale with a range of zero to five (0 – disagree strongly, 5 – agree strongly). The scale's total score ranges from 0–165, with larger scores corresponding to greater levels of fear. The W‐DEQ was translated into Persian. The validity assessment of the Persian W‐DEQ indicates that the scale consists of six factors. It showed moderate correlations with the Childbirth Attitude Questionnaire and the State‐Trait Anxiety Inventory. The Cronbach's alpha coefficients of the scale and its factors were in the acceptable range (between 0.633–0.919) (Mortazavi, 2017).

#### 3.2.4 The World Health Organization's Well‐Being Index (WHO‐5 Well‐Being Index)

The WHO‐5 Well‐Being Index comprises of five items with a six‐point Likert scale (such as “I woke up feeling fresh and rested”). Each question is scored from 0 (having good feelings at no time)–5 (having good feelings all the time). The scale's total score ranges from 0–25 which is converted to a scale of 0–100. A score of 50 has been used as the cut‐off score in screening for depression. In the case of persons with scores less than 50, referral for further assessment has been recommended. Mortazavi and colleagues investigated the validity and reliability of the Persian WHO‐5 Well‐Being Index in pregnant women. The results confirmed the reliability and unidimensionality of the scale (Cronbach's alpha = 0.85) (Mortazavi et al., 2015).

### 3.3 Data analysis

We used the SPSS version 18 to analyse the data. To characterize the sample, descriptive statistics including mean and standard deviation were used for the quantitative data and numbers and percentages for the qualitative data. We investigated the normal distribution of the birth satisfaction scores using skewness and kurtosis. We used univariate general linear model to investigate the relationships between independent variables and birth satisfaction scores. Then, all variables with a *p*‐value <.25 in the general linear models were entered into three separate multiple linear regression analyses by backward LR method. Based on these analyses, we determined the demographic/obstetric, psychological and overall predictors of birth dissatisfaction.

We checked linear regression assumptions and verified the normality of residuals. Collinearity statistics also showed tolerance <1 and variance inflation factor <2, indicating no multicollinearity. We calculated the adjusted R squared to determine the proportion of the variance of the BSS‐R scores that can be explained by the independent variables. We investigated the relationship between having a doula and birth satisfaction in emergency caesarean and vaginal birth using *t* test.

## 4 RESULTS

The mean values of age, education (in years), gestational age, birth weight and admission to delivery duration were 28.5 ± 6.1, 11.2 ± 3.7, 39.0 ± 1.3, 3170.8 ± 491.8 and 7.2 ± 7.5, respectively. The mean Wijma scores, WHO‐5 well‐being scores and birth satisfaction scores were 73.9 ± 23.0, 53.2 ± 25.2 and 23.0 ± 7.1, respectively. Fifty‐nine point six percent (59.6%) of the women were multiparous, and of these, 34.2% and 25.4% were para 2 and para 3, respectively. The correlation between birth satisfaction total scores and the duration between admissions to hospital and giving birth was −0.305 (*p* < .001). Sample characteristics are presented in Table 1. Table 1 also presents the results of general linear models for birth satisfaction scores.

**TABLE 1:** The results of general linear models on birth satisfaction scores (*N* = 767)

Demographic/obstetric variables	*N* (%)	*M* (*SD*)	Mean difference (95% CI)	*p*
Age (years)				
<20	49 (6.4)	22.4 ± 7.3	Ref	
20–30	378 (49.3)	22.8 ± 7.1	−0.409 (−1.69, 2.61)	.703
>30	340 (44.3)	23.3 ± 7.0	0.857 (−1.26, 2.97)	.427
Educational level (years)				
Primary school	125 (16.3)	22.8 ± 6.5	−0.872 (−2.46, 0.71)	.280
High school	446 (58.2)	22.8 ± 7.0	−0.888 (−2.07, 0.30)	.142
University	196 (25.6)	23.6 ± 7.5	Ref	
Job				
Housewife	690 (90.0)	22.9 ± 7.0	−1.080 (−2.74, 0.58)	.203
Employed	77 (10.0)	24.0 ± 7.2	Ref	
Household income				
Insufficient	269 (35.1)	21.7 ± 6.3	−2.005 (−3.04, −0.97)	<.001
Sufficient	498 (64.9)	23.7 ± 7.4	Ref	
Obstetric variables				
Parity				
Primipara	310 (40.4)	21.5 ± 7.0	−2.55 (−3.55, −1.55)	<.001
Multipara	457 (59.6)	24.0 ± 6.9	Ref	
History of abortion				
Yes	129 (16.8)	22.1 ± 7.1	−1.05 (−2.38, 0.29)	.123
No	638 (83.2)	23.2 ± 7.0	Ref	
Mode of birth				
Elective caesarean	101 (13.2)	24.7 ± 6.6	1.306 (−0.175, 2.79)	.084
Emergency caesarean	146 (19.0)	20.4 ± 6.9	−2.939 (−4.22, −1.66)	<.001
Vaginal delivery	520 (67.8)	23.4 ± 7.0	Ref	
Gestational age at birth (week)				
<38	104 (13.6)	21.7 ± 6.4	−2.057 (−4.01, −0.101)	.039
38–40	567 (73.9)	23.1 ± 7.1	−0.637 (−2.163, 0.888)	.412
>40	96 (12.5)	23.7 ± 7.1	Ref	
Birth weight (gr)				
<2500	66 (8.6)	21.7 ± 6.5	−1.54 (−4.56, 1.47)	.316
2500–3999	670 (87.4)	23.1 ± 7.1	−0.082 (−2.62, 2.46)	.950
≥4000	31 (4.0)	23.2 ± 7.6	Ref	
Infant gender				
Female	403 (52.5)	22.8 ± 7.0	−0.344 (−1.34, 0.66)	.500
Male	364 (47.5)	23.2 ± 7.1	Ref	
Labour pain				
Spontaneous	466 (60.8)	23.0 ± 7.1	Ref	
Induced	203 (26.5)	21.9 ± 6.9	−1.03 (−2.19, 0.125)	.080
Elective caesarean	98 (12.8)	24.8 ± 7.0	1.72 (0.19, 3.25)	.027
Having a doula				
Yes	186 (27.9)	23.9 ± 7.8	Ref	
No	480 (72.1)	22.3 ± 6.7	−1.67 (−2.85, −0.48)	.004
Elective caesarean	98 (12.8)	24.7 ± 6.6	0.748 (−0.950, 2.45)	.388
Pain relief method^a^				
Entonox	314 (60.4)	23.1 ± 7.0	1.15 (−0.20, 2.51,)	.095
Spinal/epidural analgesia	30 (5.8)	20.3 ± 5.8	3.95 (1.21, 6.70)	.005
Hot water showers/massage	23 (4.4)	24.6 ± 8.4	−0.321 (−3.40, 2.75)	.838
Nothing	153 (29.4)	24.3 ± 6.9	Ref	
Psychological variables				
Fear of childbirth				
W‐DEQ <85	529 (69.0)	25.0 ± 6.7	Ref	
W‐DEQ ≥85	238 (31.0)	18.5 ± 5.6	−6.44 (−7.42, −5.46)	<.001
WHO−5				
<50	371 (48.4)	21.3 ± 6.1	−3.37 (−4.34, −2.40)	<.001
≥50	396 (51.6)	24.6 ± 7.5	Ref	
Satisfaction with pregnancy				
Low satisfied	115 (15.0)	18.4 ± 6.2	−6.73 (−8.12, −5.33)	<.001
Moderately satisfied	281 (36.6)	22.1 ± 6.1	−3.03 (−4.06, −2.00)	<.001
Satisfied or very satisfied	371 (48.3)	25.1 ± 7.2	Ref	
Perceived marital/sexual satisfaction				
Low satisfied	11 (1.4)	18.4 ± 4.9	−4.99 (−9.16, −0.83)	.019
Moderately satisfied	69 (9.0)	20.1 ± 6.2	−3.29 (−5.01, −1.55)	<.001
Satisfied or very satisfied	687 (89.6)	23.4 ± 7.1	Ref	
Satisfaction with spouse's support				
Low satisfied	24 (3.1)	20.8 ± 7.2	−3.20 (−5.99, −0.415)	.024
Moderately satisfied	151 (19.7)	19.4 ± 5.0	−4.58 (−5.80, −3.36)	<.001
Satisfied or very satisfied	592 (77.2)	24.0 ± 7.2	Ref	

Abbreviation: GLM, general linear model.

^a^ NVD group was included in the analysis.

In Table 2, detailed results of multiple linear regression analysis for the birth satisfaction scores are presented. Predictors of low birth satisfaction were primiparity [Beta = −0.131, CI (−2.745, −1.021)], low level of well‐being [−0.119, (−2.514, −0.844)], low [−0.193 (−5.052, −2.568)] and moderate [−0.143 (−2.999, −1.199)] satisfaction with pregnancy, moderate satisfaction with spouse's emotional/financial support [−0.150 (−3.718,‐1.595)], emergency caesarean [−0.086 (−2.713, −0.360)], severe fear of childbirth [−0.315 (−5.701, −3.911)] and long interval between admission to hospital and giving birth [−0.188 (−0.233, −0.119)].

**TABLE 2:** Results of multiple linear regression analysis on the birth satisfaction scores

	Unstandardized Coefficients		Standardized Coefficients		95.0% CI for B	
	B	S.E	Beta	*p*	Lower Bound	Lower Bound
Demographic/obstetric predictors						
Contrast	28.606	0.802		<.001	27.032	30.181
Insufficient income	−2.027	0.504	−0.137	<.001	−3.017	−1.037
Primiparity	−1.596	0.504	−0.111	.002	−2.586	−0.606
Emergency caesarean	−1.850	0.640	−0.103	.004	−3.108	−0.593
Spinal/epidural	−2.123	1.236	−0.058	.086	−4.550	0.303
Not having a doula	−0.897	0.514	−0.062	.081	−1.905	0.112
Long admission To delivery duration	−0.233	0.033	−0.248	<.001	−0.298	−0.167
Psychological predictors						
Contrast	32.668	0.678		<.001	31.337	33.999
Low satisfaction with pregnancy	−4.247	0.665	−0.215	<.001	−5.552	−2.942
Moderate satisfaction with pregnancy	−1.912	0.484	−0.131	<.001	−2.863	−0.962
Moderate satisfaction with spouse's support	−2.745	0.560	−0.155	<.001	−3.843	−1.646
Severe fear of childbirth	−5.298	0.480	−0.348	<.001	−6.240	−4.356
Low level of well‐being	−1.775	0.449	−0.126	<.001	−2.656	−0.895
Overall predictors						
Contrast	36.875	0.890		<.001	35.128	38.622
Insufficient income	−0.803	0.440	−0.054	.069	−1.667	0.061
Primiparity	−1.883	0.439	−0.131	<.001	−2.745	−1.021
Low level of well‐being (ref: WHO−5 ≥ 50)	−1.679	0.425	−0.119	<.001	−2.514	−0.844
Low satisfaction with pregnancy (ref: high satisfaction)	−3.810	0.633	−0.193	<.001	−5.052	−2.568
Moderate satisfaction with pregnancy (ref: high satisfaction)	−2.099	0.458	−0.143	<.001	−2.999	−1.199
Moderate satisfaction with spouse's support (ref: high satisfaction)	−2.657	0.541	−0.150	<.001	−3.718	−1.595
Emergency caesarean (ref: vaginal birth)	−1.536	0.599	−0.086	.011	−2.713	−0.360
Severe fear of childbirth (ref: low fear)	−4.806	0.456	−0.315	<.001	−5.701	−3.911
Long admission To delivery duration	−0.176	0.029	−0.188	<.001	−0.233	−0.119

Note

R square for the first regression analysis = 14.5%, R square for the second regression analysis = 28.8%, R square for the third regression analysis = 36.8%, method: backward.

We looked into the relation between the presence of a doula during labour and delivery and birth satisfaction in the case women who had emergency caesarean and also women who had vaginal delivery. Women who had vaginal birth and were accompanied by a doula reported a higher level of birth satisfaction than those without a doula (*p* = .012). Women who had emergency caesarean accompanied by a doula reported a lower level of birth satisfaction than those without a doula (*p* = .045) (Table 3).

**TABLE 3:** Distribution of the birth satisfaction scores according to the mode of birth and having a Doula at birth (*N* = 666)

Having a doula at birth	Emergency Caesarean		Vaginal birth	
	*N*	Mean ± *SD*	*N*	Mean ± *SD*
No	131	20.8 ± 7.0	349	22.8 ± 6.6
Yes	15	17.1 ± 5.3	171	24.5 ± 7.7
t	2.02		2.52	
P	0.045		0.012	

## 5 DISCUSSION

In this study, we investigated the predictors of low birth satisfaction in early postpartum. Our results indicate that primiparity, emergency caesarean, long duration from admission to delivery, low level of well‐being, low and moderate satisfaction with pregnancy, moderate satisfaction with spouse's emotional/financial support and severe fear of childbirth are predictors of low birth satisfaction. The proportion of the variance in birth satisfaction explained by the demographic and psychological variables was 14.5% and 28.8%, respectively. The total explained variance by all the independent variables was 36.8%. Our results can be compared to those obtained by Nahaee and colleagues in a study in Tehran which investigated, pre‐ and during‐labour predictors of low birth satisfaction. They found that the proportion of the variance explained by during‐labour, pre‐labour and overall variables was 75%, 14% and 76%, respectively (Nahaee et al., 2020). In our study, the highest proportion (28.8%) of variation in birth satisfaction scores is explained by the psychological variables, which include fear of childbirth, well‐being, satisfaction with pregnancy and satisfaction with spouse's emotional/financial support.

We found that postpartum fear of childbirth was a predictor of birth satisfaction. This result is in agreement with those reported by Nahaee et al. in Tehran, Iran (Nahaee et al., 2020). A survey on 1004 Indian women found that experiencing no fear of childbirth had a positive impact on birth satisfaction in women who gave birth vaginally (Jha et al., 2017). In our study, women's low level of well‐being was a predictor of low birth satisfaction. We used the WHO‐5 Well‐Being Index which was recommended as a depression screening tool ("About the WHO‐5.", 2020). This result is consistent with that of a previous study which found severe anxiety state was a predictor of low birth satisfaction (Nahaee et al., 2020).

In our study, having a smooth, hassle‐free pregnancy was a predictor of birth satisfaction. In a study on 225 postpartum women in Khaf, Iran, childbirth experience improved with lower hassle and an increased sense of uplift (Khalife‐Ghaderi et al., 2021). In our study, satisfaction with spouse's support could predict birth satisfaction. In a study on 652 women at 12 to 48 hr postpartum, helpfulness of partner support was a predictor of a satisfactory birth experience (Bryanton et al., 2008).

Results from this study indicate that primiparity is a predictor of low birth satisfaction. This is in agreement with results from studies by Nahaee et al. (Nahaee et al., 2020), Takacs et al. (Takacs et al., 2015) and Fumagalli et al. (Fumagalli et al., 2021). Results from our study and similar studies indicate that in primiparous women, childbirth experience is associated with more fear in comparison with multiparous and that labour duration is usually longer in nulliparous than in multiparous. Both of these facts may have a role in low birth satisfaction. In our study, long labour duration was a strong predictor of low birth satisfaction, which is in line with other studies conducted in Canada (Bélanger‐Lévesque et al., 2014), Italy (Fumagalli et al., 2021) and Ethiopia (Demis et al., 2020). Dystocia was also a predictor of low satisfaction in Nahaee et al.’s study (Nahaee et al., 2020).

We found that emergency caesarean could predict low birth satisfaction. In two studies on postpartum women in Canada (Bryanton et al., 2008) and Ethiopia (Bitew et al., 2015), type of birth was the strongest predictor of women's childbirth perceptions. However, the result of a study conducted in Iran showed no relationship between the mode of childbirth and level of satisfaction (Spaich et al., 2013).

We found no socio‐demographic variables related to maternal satisfaction which is in line with Fumagalli et al.’s study (Fumagalli et al., 2021). But in a study conducted in Italy, women with higher levels of educational attainment had a higher level of vaginal birth satisfaction than women with lower levels of education (Tocchioni et al., 2018). In contrast to the above study, two other studies found that women with low educational levels (Asres, 2018) or no formal education (Tadele et al., 2020) had higher levels of birth satisfaction.

Our results indicate that having a doula at birth is one of the predictors of birth satisfaction. Further investigation showed that the effect of having a doula during labour and birth on birth satisfaction depends on the mode of birth. In women who eventually had a vaginal delivery, having a companion had a significant positive effect on birth satisfaction, but in women who had an emergency caesarean, it had a significant negative effect. This result is in line with previous studies that found that in normal vaginal deliveries, having a doula at birth improved maternal emotional well‐being, reduced anxiety and fear of childbirth (McLeish & Redshaw, 2019; Mortazavi & Mehrabadi, 2021). In addition, evidence from other studies indicates that presence of a companion at birth is a predictor of childbirth experience (Khalife‐Ghaderi et al., 2021) and increases the overall birth satisfaction (Mocumbi et al., 2019). Since the implementation of the HTP in Iran, free childbirth preparatory classes are regularly organized for pregnant women. Participants in these classes are allowed to have a doula present during labour and childbirth. Our results indicate that women who pay to have a doula during normal vaginal birth but fail to deliver normally, experience a sense of frustration which impacts on their satisfaction with childbirth. Such cases should be considered by public health officials who are developing programs and interventions which aim to improve childbirth satisfaction in early postpartum.

We found that receiving spinal/epidural analgesic is not a predictor of birth satisfaction. In one study, administration of analgesics during labour was found to be associated with birth satisfaction (Dickinson et al., 2003), while in another study, no intrapartum intervention (Fumagalli et al., 2021) was associated with birth satisfaction.

The mean birth satisfaction score was 23.0 ± 7.1, which is close to that reported in Nahaee et al.’s study in Tehran, Iran (Nahaee et al., 2020) and Goncu et al.’s study in Turkey (Goncu Serhatlioglu et al., 2018). In their studies, the mean birth satisfaction scores were 23.8 ± 6.5 and 20.4 ± 6.0, respectively. The birth satisfaction mean score in our study is lower in comparison with studies conducted in developed countries such as the USA (32.4 ± 6.4) (Martin, Hollins Martin, Burduli, et al., 2017), Australia (30.4 ± 5.9) (Jefford et al., 2018) and UK (28.4 ± 5.6) (Martin & Martin, 2014).

Due to the increased Internet and satellite TV penetration in Iran, women have access to more information about the higher quality of maternity services and better conditions of childbirth in developed countries. This may have led to higher levels of expectations about childbirth. The mismatch between expectations and available maternity services and substandard care may have a role in lower birth satisfaction in developing countries (Mortazavi & Mirzaii, 2012). Moreover, in developed countries, the evolution of maternity services has led to a shift in the focus of health policy towards the provision of woman‐centred individualized care (Martin et al., 2017), but in developing countries, with the reduction in maternal mortality and morbidity, the focus of policy has only recently shifted to improving birth satisfaction.

### 5.1 Limitations and strength

In this cross‐sectional study, we measured fear of childbirth and birth satisfaction in early postpartum at the same time; therefore, the cause‐and‐effect relationship between these two variables is difficult to determine although it seems more plausible to assume that the direction of causality is from fear of childbirth to birth satisfaction. We investigated birth satisfaction during the first 24 hr postpartum. It must be noted that after discharge from hospital, factors such as substandard or discontinued maternity care may reduce satisfaction with childbirth. This in turn may adversely impact perceived overall birth satisfaction. Also, some other factors of interest such as domestic violence were not investigated in this study. The strong point of this study is the large sample size which enabled us to conduct multiple regression analysis with several significant variables. This study was conducted on a convenience sample of postpartum women. Its findings cannot be generalized to populations with characteristics that differ from the study population.

### 5.2 Implications for future research

We recommend that further studies be conducted in Iran to identify other significant factors influencing birth satisfaction, particularly social predictors of birth satisfaction such as helpfulness of midwives/physicians, communication of information and respectful attitude of staff members. Also, attitudes and expectations of parturient women with regard to participation in decision‐making and having choice and autonomy during labour and birth should be investigated. Furthermore, the trajectory of birth satisfaction during the first two weeks postpartum may reveal the effects of other important variables on birth satisfaction such as breastfeeding problems.

## 6 CONCLUSIONS

Our findings indicate that the obstetric/psychological predictors of birth satisfaction are responsible for 36.8% of variation in birth satisfaction. We found that primiparity, emergency caesarean, long duration between admission and delivery, low level of well‐being, low and moderate satisfaction with pregnancy, moderate satisfaction with spouse's support and severe fear of childbirth were the predictors of the low birth satisfaction. Policies can be designed to influence some of these factors with the aim enhancing women's satisfaction with the experience of childbirth. The following are some of the measures which should be considered in this regard: interventions to reduce antepartum and intrapartum fear of childbirth, measures which facilitate spouse's support during pregnancy and childbirth, provision of free doula care and appropriate changes to hospital admission procedures to prevent long stays in the labour room (e.g. provision of a space for parturients who would be closely tended before being hospitalized).

## CONFLICT OF INTERESTS

The authors have no conflict of interest.

## AUTHOR CONTRIBUTIONS

MM collected the data and wrote the first draft of the manuscript. FM analysed the data and wrote the final draft of the manuscript. The authors have read and approved the manuscript.

## ETHICS APPROVAL AND CONSENT FOR PARTICIPATION IN RESEARCH

The Ethics Committee of Sabzevar University of Medical Sciences has reviewed and approved this study (approval number: IR.MEDSAB.REC.1400.119). All procedures were performed in accordance with the guidelines of Sabzevar University of Medical Sciences, which is in accordance with the Declaration of Helsinki. Women who consented to participate in the study signed an informed consent form and were assured about the confidentiality of their information.

## Supporting information

Supplementary Material

## Data Availability

The data that support the findings of this study are available from the corresponding author, upon reasonable request.
