# Supplementary material for: Predictors of low birth satisfaction among Iranian postpartum women: A cross‐sectional study
Source: Nurs Open. 2021 Oct 30;9(1):604–13. doi: 10.1002/nop2.1104 (PMC8685877; doi:10.1002/nop2.1104)
Supplement: Supplementary file 1 — Supplementary Material [file NOP2-9-604-s001.docx]

Table1s. The percentage of the Farsi BSS-R items

|  |  |  | Not at all |  |  |  | Strongly agree |
| --- | --- | --- | --- | --- | --- | --- | --- |
| Factors | Item number |  | 0 | 1 | 2 | 3 | 4 |
| Stress | BSS-R 1 | I came through childbirth virtually unharmed | 7.3 | 9.5 | 19.8 | 28 | 35.4 |
|  | BSS-R 2 | I thought my labor was excessively long | 35.5 | 16.7 | 18.2 | 14.6 | 15.2 |
|  | BSS-R 3 | The birthing room staff encouraged me to make decisions about how I wanted my birth to progress | 17.1 | 15.4 | 21.2 | 16.7 | 29.6 |
|  | BSS-R 4 | I felt very anxious during my labor and birth | 49 | 15.7 | 17.3 | 10.4 | 7.6 |
| Attributes | BSS-R 5 | I felt well supported by staff during my labor and birth | 2.5 | 4.5 | 17.3 | 29.1 | 46.6 |
|  | BSS-R 6 | The staff communicated well with me during labor | 1.3 | 4.7 | 15.4 | 32.1 | 46.6 |
| Quality | BSS-R 7 | I found giving birth a distressing experience | 25.5 | 20.9 | 22.7 | 12.4 | 18.4 |
|  | BSS-R 8 | I felt out of control during my birth experience | 19 | 20.1 | 22.1 | 16.3 | 22.5 |
|  | BSS-R 9 | I was distressed during labor | 31.9 | 21.8 | 23.6 | 13.6 | 9.1 |
|  | BSS-R 10 | The delivery room was clean and hygienic | 0.4 | 2.5 | 12.3 | 27.8 | 57 |
| Total (10 items) | |  |  |  |  |  |  |

Table 2. Comparison of Iranian BSS-R total and sub-scale scores differentiated by parity. Standard deviations are in parentheses, degrees of freedom = 782, CI = confidence interval.

| BSS-R Scale | Primip (N=310) | Multip (N=457) | 95% CI | *t* | *p* | Hedges g | 95% CI | Effect size |
| --- | --- | --- | --- | --- | --- | --- | --- | --- |
| Stress | 6.85 (3.81) | 8.36 (3.82) | 0.96 – 2.05 | 5.42 | <0.001 | 0.39 | 0.25 – 0.54 | Small |
| Attributes | 2.89 (2.17) | 3.40 (2.31) | 0.19 – 0.83 | 3.10 | 0.002 | 0.23 | 0.08 – 0.37 | Small |
| Quality | 11.64 (3.26) | 12.25 (3.01) | 0.17 – 1.06 | 2.72 | 0.007 | 0.20 | 0.05 – 0.34 | Negligible |
| Total score | 21.38 (6.95) | 24.01 (6.90) | 1.64 – 3.62 | 5.23 | <0.001 | 0.40 | 0.24 – 0.52 | Small |

Table3s. Participants' characteristics and correlation with F-BSS-R total scores

|  | Mean(SD) | R | P |
| --- | --- | --- | --- |
| Age (year) | 28.43(6.07) | 0.042 | 0.243 |
| Education (year) | 11.22(3.65) | 0.029 | 0.416 |
| Admission To Delivery Duration (hour) | 7.58(13.72) | **-0.305** | **<0.001** |
| Labor Duration (hour) | 4.59(8.22) | -0.016 | 0.654 |
| Gestational age (week) | 39.01(1.32) | 0.046 | 0.196 |
| Birth Weight (gram) | 3173.53(490.78) | 0.059 | 0.097 |

Table4s. The means of the Farsi W-DEQ factors.

|  | | Lack of Self-efficacy | Lack of positive anticipation | Loneliness | Fear | Fear of harm to child | Fear of Losing control |
| --- | --- | --- | --- | --- | --- | --- | --- |
| N | Valid | 767 | 767 | 767 | 767 | 767 | 767 |
|  | Missing | 0 | 0 | 0 | 0 | 0 | 0 |
| Mean | | 23.32 | 5.50 | 18.71 | 15.22 | 3.24 | 5.31 |
| Std. Deviation | | 11.52 | 4.05 | 9.48 | 5.17 | 3.51 | 3.15 |
| Minimum | | .00 | .00 | .00 | .00 | .00 | .00 |
| Maximum | | 50.00 | 20.00 | 40.00 | 25.00 | 10.00 | 15.00 |
